# The prognostic value of the tumor–stroma ratio is most discriminative in patients with grade III or triple‐negative breast cancer

**DOI:** 10.1002/ijc.32857

**Published:** 2020-01-22

**Authors:** Kiki M.H. Vangangelt, Andrew R. Green, Isabelle M.F. Heemskerk, Danielle Cohen, Gabi W. van Pelt, Marcelo Sobral‐Leite, Marjanka K. Schmidt, Hein Putter, Emad A. Rakha, Rob A.E.M. Tollenaar, Wilma E. Mesker

**Affiliations:** ^1^ Department of Surgery Leiden University Medical Center Leiden The Netherlands; ^2^ Nottingham Breast Cancer Research Center, Division of Cancer and Stem Cells, School of Medicine The University of Nottingham, Nottingham City Hospital Nottingham United Kingdom; ^3^ Department of Pathology Leiden University Medical Center Leiden The Netherlands; ^4^ Division of Molecular Pathology Netherlands Cancer Institute Amsterdam The Netherlands; ^5^ Department of Biomedical Data Sciences Leiden University Medical Center Leiden The Netherlands

**Keywords:** tumor–stroma ratio, breast cancer, prognosis, grade, triple‐negative status

## Abstract

The tumor–stroma ratio (TSR) was evaluated as a promising parameter for breast cancer prognostication in clinically relevant subgroups of patients. The TSR was assessed on hematoxylin and eosin‐stained tissue slides of 1,794 breast cancer patients from the Nottingham City Hospital. An independent second cohort of 737 patients from the Netherlands Cancer Institute to Antoni van Leeuwenhoek was used for evaluation. In the Nottingham Breast Cancer series, the TSR was an independent prognostic parameter for recurrence‐free survival (RFS; HR 1.35, 95% CI 1.10–1.66, *p* = 0.004). The interaction term was statistically significant for grade and triple‐negative status. Multivariate Cox regression analysis showed a more pronounced effect of the TSR for RFS in grade III tumors (HR 1.89, 95% CI 1.43–2.51, *p* < 0.001) and triple‐negative tumors (HR 1.86, 95% CI 1.10–3.14, *p* = 0.020). Comparable hazard ratios and confidence intervals were observed for grade and triple‐negative status in the ONCOPOOL study. The prognostic value of TSR was not modified by age, tumor size, histology, estrogen receptor status, progesterone receptor status, human epidermal growth factor receptor 2 status or lymph node status. In conclusion, patients with a stroma‐high tumor had a worse prognosis compared to patients with a stroma‐low tumor. The prognostic value of the TSR is most discriminative in grade III tumors and triple‐negative tumors. The TSR was not modified by other clinically relevant parameters making it a potential factor to be included for improved risk stratification.

## Abstract 2

**What's new?**

A tumor does not exist in isolation, and evaluation of its microenvironment or “stroma” could help determine clinical outcome in breast cancer. Here, the authors show that women with a stroma‐high tumor had a worse prognosis compared to women with a stroma‐low tumor. The prognostic value of the tumor‐stroma ratio was most discriminative in patients with grade III or triple negative breast cancer, thus making it a potential valuable factor to improve risk stratification for these patients.

**Abbreviations**
BCSS: breast cancer‐specific survival
CAFs: cancer‐associated fibroblasts
CI: confidence interval
ER: estrogen receptor
H&E: hematoxylin and eosin
HER2: human epidermal growth factor 2
HR: hazard ratio
NST: no special type
OS: overall survival
PR: progesterone receptor
RFS: recurrence‐free survival
TSR: tumor–stroma ratio

## Introduction

Breast cancer mortality rates are declining in most European countries due to early detection and improved treatment options.^1^ Optimizing risk stratification to prevent undertreatment and overtreatment by personalizing therapy is thereby essential.

In the last decade, the interplay of tumor cells and its microenvironment has gained increased interest. The tumor microenvironment, also known as tumor‐associated stroma, consists of immune cells, fibroblasts, pericytes and endothelial cells in an extracellular matrix. The tumor microenvironment plays an active role in creating an environment that favors the tumor cells; increased motility of cells, suppression of the immune response, remodeling of the extracellular matrix and angiogenesis.^2^, ^3^, ^4^, ^5^, ^6^

A promising prognostic parameter based on the tumor‐associated stroma is the tumor–stroma ratio (TSR). The TSR reflects the amount of tumor–stroma to the cancer cells, which is determined on routinely retrieved hematoxylin and eosin (H&E) stained tissue slides used for pathological assessment of surgically removed breast tissue. TSR assessment is easy, quick and without additional costs. Previous research demonstrated the prognostic value of the TSR in different types of invasive solid tumors, including breast cancer.^7^, ^8^, ^9^, ^10^, ^11^, ^12^, ^13^, ^14^, ^15^, ^16^, ^17^, ^18^, ^19^, ^20^, ^21^, ^22^, ^23^, ^24^, ^25^, ^26^, ^27^, ^28^, ^29^, ^30^, ^31^, ^32^ Most of these studies validated a worse prognosis for patients with stroma‐high tumors.

Breast cancer is a heterogeneous disease, which makes subgroup analysis essential. Kramer *et al*. reviewed literature published on the prognostic value of TSR in the general breast cancer population and different clinically important subgroups.^33^ Here, we set out to validate the effect of the TSR and further expand its utility in the clinically relevant subgroups for breast cancer prognostication. This is an essential step toward prospective validation and clinical implementation, such as the addition of TSR to the frequently used online prediction tool PREDICT.

## Materials and Methods

### Study population

#### *The Nottingham breast cancer series from Nottingham City Hospital (UK)*

The study population consists of women of ≤70 years with primary invasive breast cancer without distant metastases, diagnosed and treated primarily with surgery in the Nottingham City Hospital between 1993 and 2002 (*n* = 1809). This cohort was retrospectively assembled. Patients were included if digital H&E slides of the primary breast tumors and follow to up data were available. Exclusion criteria were breast cancer in premedical history and/or neoadjuvant treatment.

#### *The ONCOPOOL study from the Netherlands Cancer Institute to Antoni van Leeuwenhoek (the Netherlands)*

A total of 737 women treated primarily with surgery for invasive nonmetastatic breast cancer between 1990 and 1999, included in the ONCOPOOL study at The Netherlands Cancer Institute‐Antoni van Leeuwenhoek hospital, were analyzed in our study. The included patients were part of the larger ONCOPOOL database of European primary breast cancer patients. Details on data management and patient selection were described previously.^14^, ^34^ Survival data, estrogen receptor (ER) status, progesterone receptor (PR) status are updated since the previous publication on TSR according to the last publication using the ONCOPOOL study.^14^, ^35^

All patient data were used in an anonymized manner and data were handled according to national ethical guidelines (“Code for Proper Secondary Use of Human Tissue”, Dutch Federation of Medical Scientific Societies”). No additional informed consent was required. The Nottingham Breast Cancer Series was approved by the Nottingham Research Ethics Committee 2 under the title “Development of molecular genetic classification of breast cancer.”

### Assessment of the TSR

In the Nottingham Breast Cancer series, the TSR was visually assessed on digital H&E stained slides of the primary breast tumor *via* CaseViewer 2.2 for Windows (3DHISTECH Ltd., Budapest, Hungary), a digital application for the evaluation of microscopic images. The original 4 μm routine H&E stained slides were scanned into high‐resolution (0.19 μm/pixel) digital images at 20× magnification using 3DHistech Panoramic 250 Flash II scanner (3DHISTECH Ltd., Budapest, Hungary). First, the whole tissue slide was visually evaluated for the orientation of the most stromal rich field. Second, the most stromal abundant area was annotated using a circle with an area of 3.1 mm^2^. This microscopic field is comparable with the surface selected with a 10× objective of most light microscopes and corresponds with the magnification used in previously.^36^ All slides were double scored in a blinded fashion (KV,WM). A third observer (DC) was consulted if consensus could not be reached. The tissue slide with the highest stroma percentage was decisive in cases where multiple slides were available per patient. Stromal areas suspected of postbiopsy effects were excluded from TSR assessment.

The TSR assessment on tumor tissue of patients included in the ONCOPOOL study was assessed using visual microscopy on conventional H&E slides.^14^

The TSR was scored by the method of Mesker *et al*. in both cohorts.^7^ A percentage of ≤50% stroma was categorized as stroma‐low and >50% stroma was categorized as stroma‐high (Supporting Information Fig. S1).

### Statistical analyses

Statistical analyses were performed using IBM SPSS statistics (version 23 for Windows). The recurrence‐free survival (RFS), the primary endpoint, was defined as the time between the date of diagnosis and local, regional or distant recurrence. Patients who died without a recurrence were censored. Breast cancer‐specific survival (BCSS), the secondary endpoint in the Nottingham Breast Cancer series, was defined as the time from date of diagnosis and breast cancer‐specific death. The BCSS was not available for the ONCOPOOL study. Therefore, in this cohort, the overall survival (OS) was used as the second endpoint. The OS was defined as the time from diagnosis to death from any cause.

The *X* ^*2*^ test was used to evaluate the difference between categorical variables in stroma‐low and stroma‐high groups. Fisher's exact test was performed if less than five patients were included per category and Fisher–Freeman–Halton when the table was larger than 2 × 2. The Kaplan–Meier method and the log‐rank test were performed. Cohen's kappa coefficient was used to test interobserver variability.

The Cox regression model was used to perform univariate and multivariate analysis. In the multivariate Cox regression analysis of the Nottingham Breast Cancer series, the TSR and confounders were entered; age at diagnosis (continuous), grade (I, II or III), size (≤2 cm and >2 cm), histological type (invasive carcinoma of no special type [NST], lobular carcinoma, tubular carcinoma and others), ER status (negative or positive), PR status (negative or positive) and human epidermal growth factor receptor 2 (HER2) status (negative or positive). These analyses were also performed with triple‐negative status as a variable instead of ER status, PR status and HER2 status. Also, lymph node status was entered in the multivariate Cox regression in addition to standard confounders as described above, as lymph node status is not a confounder but a clinically important parameter. A *p*‐value <0.05 was considered as statistically significant. The univariate and multivariate Cox regression analysis of the ONCOPOOL study were also performed as described in the original report of Roeke *et al*., to check reproducibility. For the evaluation of the prognostic value of the TSR for clinically relevant subgroups, the interaction term was introduced in the Cox regression analysis. This was corrected for clinically relevant confounders as described above.

## Results

### Patients

#### *The Nottingham breast cancer series*

A total of 2,385 H&E slides of 1,809 patients were assessed for TSR. The slides of 15 (0.8%) patients were not eligible for TSR scoring due to the poor quality of the tissue. The Cohen's kappa coefficient was 0.61 between two observers, which corresponds with a substantial to a good level of agreement. Due to the digital learning curve, slides with an incongruent value were individually assessed by the same observers for a second time (blinded from their first scores). Cohen's kappa coefficient in the total cohort increased up to 0.87, which corresponds with an almost perfect level of agreement. The H&E slides of 37 patients were discussed with a third observer. A final agreement for the TSR was reached in all cases. A total of 1,794 patients were suitable for statistical analysis. The median age at the time of diagnosis was 55 years (range 23–70 years), and the median follow‐up period was 11 years (range 0–18 years). Table 1 provides an overview of patient and tumor characteristics.

**Table 1:** Overview of the stratification of age and tumor characteristics of the patients included in the Nottingham Breast Cancer Series

		Stroma‐low		Stroma‐high		
	*n*	*n* = 681	%	*n* = 1,113	%	*p*‐value
*Age (in years)*						
<40	144	71	10.4	73	6.6	0.006
40 to <50	385	151	22.2	234	21.0	
50 to <60	636	247	36.3	389	35.0	
≥60	628	212	31.1	416	37.4	
Missing	1	0	0.0	1	0.1	
*Tumor size (in cm)*						
≤2	1,146	505	74.2	641	57.6	<0.001
>2 to <5	625	169	24.8	456	41.0	
≥5	21	6	0.9	15	1.3	
Missing	2	1	0.1	1	0.1	
*Lymph node involvement*						
No	1,127	452	66.4	675	60.6	0.015
Yes	664	227	33.3	437	39.3	
Missing	3	2	0.3	1	0.1	
*Grade*						
I	279	105	15.4	174	15.6	0.606
II	733	272	39.9	461	41.4	
III	781	303	44.5	478	42.9	
Missing	1	1	0.1	0	0	
*Histological type*						
Invasive carcinoma of NST	1,129	450	66.1	679	61.0	0.117
Lobular carcinoma	155	53	7.8	102	9.2	
Tubular carcinoma	275	90	13.2	185	16.6	
Others	235	88	12.9	147	13.2	
*ER status*						
Negative	331	151	22.2	180	16.2	0.001
Positive	1,463	530	77.8	933	83.8	
*PR status*						
Negative	708	282	41.4	426	38.3	0.262
Positive	1,067	390	57.3	677	60.8	
Missing	19	9	1.3	10	0.9	
*HER2 status*						
Negative	1,573	594	87.2	979	88.0	0.645
Positive	221	87	12.8	134	12.0	
*Triple‐negative tumors*						
No	1,546	560	82.2	986	88.5	0.001
Yes	235	115	16.9	120	10.8	
Missing	13	6	0.9	7	0.6	
*Chemotherapy*						
No	699	255	37.4	444	39.9	0.577
Yes	292	115	16.9	177	15.9	
Missing	803	311	45.7	492	44.2	
*Hormonal therapy*						
No	455	182	26.7	273	24.5	0.112
Yes	778	274	40.2	504	45.3	
Missing	561	225	33.0	336	30.2	

Abbreviations: ER, estrogen receptor; HER2, human epidermal growth factor receptor 2; NST, no special type; PR, progesterone receptor.

#### *The ONCOPOOL study*

The ONCOPOOL study included 737 women with breast cancer and was previously analyzed for the prognostic value of the TSR.^14^ The median age at inclusion was 54 (range 23–71 years). The median follow‐up was 12 years (range 0–24 years). Patient, tumor and treatment characteristics are shown in Supporting Information Table S1.

### The prognostic value of the TSR

In the total study population of the Nottingham Breast Cancer series, 681 (38%) patients were categorized in the stroma‐low group and 1,113 (62%) patients in the stroma‐high group. Table 1 shows the statistically significant differences between both stroma categories. Age, tumor size, lymph node involvement, ER status and triple‐negative tumors were significantly different between both stromal categories.

The Kaplan–Meier analysis and the log‐rank test for RFS showed a statistically significant different outcome between patients with a stroma‐low and stroma‐high tumor in favor of patients with stroma‐low tumors (Supporting Information Fig. S2). The TSR was an independent prognostic parameter in favor of patients with stroma‐low tumors for both RFS and BCSS when adjusted for different sets of confounders (Tables 2 and 3).

**Table 2:** Univariate and multivariate analysis of the Nottingham Breast Cancer Series calculated by Cox regression analysis

		Recurrence‐free survival						Breast cancer‐specific survival					
		Univariate analysis			Multivariate analysis			Univariate analysis			Multivariate analysis		
	*n*	HR	95% CI	*p*‐value	HR	95% CI	*p*‐value	HR	95% CI	*p*‐value	HR	95% CI	*p*‐value
*Age*													
	1,793	0.99	1.98–1.00	0.129	1.00	0.99–1.01	0.680	0.99	0.98–1.00	0.222	1.01	0.99–1.02	0.388
*Tumor size (in cm)*													
≤2	1,146			<0.001			<0.001			<0.001			<0.001
>2	646	2.17	1.81–2.61		1.73	1.42–2.11		2.41	1.93–3.00		1.70	1.35–2.15	
*Grade*													
I	279			<0.001			<0.001			<0.001			<0.001
II	733	1.52	1.07–2.15		1.24	0.83–1.84		2.96	1.66–5.29		2.32	1.34–4.37	
III	781	2.91	2.09–4.06		2.08	1.37–3.15		7.01	4.01–12.28		4.67	2.46–8.88	
*Histological type*													
Invasive carcinoma of NST	1,129			<0.001			0.012			<0.001			0.083
Lobular carcinoma	155	0.93	0.67–1.30		1.30	0.91–1.85		0.90	0.61–1.32		1.44	0.94–2.19	
Tubular carcinoma	275	0.53	0.38–0.72		0.98	0.70–1.43		0.34	0.22–0.53		0.93	0.56–1.55	
Others	235	1.16	0.90–1.50		1.51	1.16–1.97		0.98	0.72–1.34		1.41	1.02–1.95	
*ER status*													
Negative	331			<0.001			0.296			<0.001			0.801
Positive	1,463	0.64	0.52–0.80		1.16	0.88–1.54		0.50	0.39–0.63		1.04	0.76–1.43	
*PR status*													
Negative	708			<0.001			0.011			<0.001			0.005
Positive	1,067	0.61	0.51–0.73		0.74	0.59–0.93		0.50	0.40–0.62		0.67	0.51–0.89	
*HER2 status*													
Negative	1,573			<0.001			<0.001			<0.001			0.002
Positive	221	2.09	1.66–2.64		1.70	1.32–2.18		2.27	1.74–2.96		1.56	1.17–2.08	
*TSR*													
Stroma‐low	681			<0.001			0.004			<0.001			0.001
Stroma‐high	1,113	1.46	1.19–1.78		1.35	1.10–1.66		1.60	1.25–2.04		1.51	1.18–1.95	

Abbreviations: ER, estrogen receptor; HER2, human epidermal growth factor receptor 2; NST, no special type; PR, progesterone receptor; TSR, tumor–stroma ratio.

**Table 3:** Results of the independent prognostic value of the tumor–stroma ratio adjusted for only confounders or confounders combined with triple‐negative status or lymph node status calculated with multivariate Cox regression analysis in the Nottingham Breast Cancer series

	Recurrence‐free survival	Breast cancer‐specific survival
Confounders	HR 1.35, 95% CI 1.10–1.66, *p* = 0.004	HR 1.51, 95% CI 1.18–1.95, *p* = 0.001
Confounders including triple‐negative status	HR 1.34, 95% CI 1.09–1.64, *p* = 0.006	HR 1.47, 95% CI 1.15–1.90, *p* = 0.002
Confounders and lymph node status	HR 1.35, 95% CI 1.10–1.66, *p* = 0.004	HR 1.51, 95% CI 1.17–1.94, *p* = 0.002

Since the ONCOPOOL study was updated, the prognostic value of the TSR was evaluated again. The analyses showed that patients with a tumor with a high stromal content had a worse survival, in the total cohort as well as in subgroups. The results from the multivariate Cox regression analysis of the updated database were comparable with those of the original observations; RFS HR 1.35, 95% CI 1.01–1.79, *p* = 0.040 *versus* HR 1.35, 95% CI 1.01–1.81, *p* = 0.046 and OS HR 1.46, 95% CI 1.13–1.88, *p* = 0.003 *versus* HR 1.56, 95% CI 1.18–2.05, *p* = 0.002, respectively (data not shown). When the TSR was adjusted for confounders, the OS showed a statistically significant difference in favor of stroma‐low tumors. The results for the RFS were borderline statically significant (Supporting Information Tables S2 and S3).

### TSR stratified by clinically important subgroups

In Cox regression analysis, the interaction term was introduced, to evaluate the prognostic effect in different clinically important subgroups.

**Figure 1:**
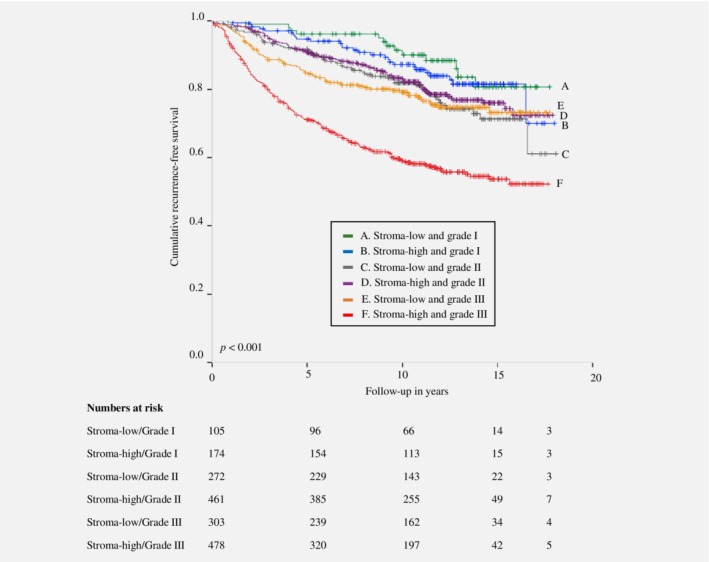
Kaplan–Meier analysis for recurrence‐free survival of patients included in the Nottingham Breast Cancer Series stratified by tumor–stroma ratio combined with grade. [Color figure can be viewed at http://wileyonlinelibrary.com]

**Figure 2:**
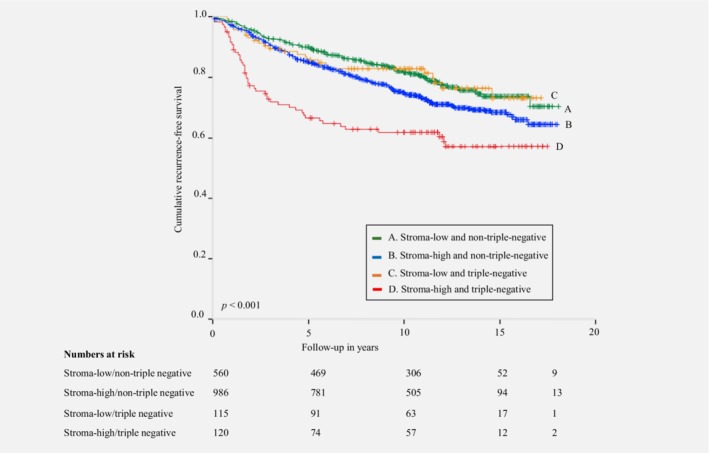
Kaplan–Meier analysis for recurrence‐free survival of patients included in the Nottingham Breast Cancer Series stratified by tumor–stroma ratio combined with triple‐negative status. [Color figure can be viewed at http://wileyonlinelibrary.com]

In the Nottingham Breast Cancer series, the interaction term showed a statistically significant *p*‐value for grade (*p* < 0.001 and *p* = 0.002) and triple‐negative status (*p* = 0.040 and *p* = 0.026) for RFS and BCSS, respectively. No statistically significant results for RFS and BCSS were observed if stratified for age, tumor size, histology, ER status, PR status, HER2 status and lymph node status. The prognostic value of the TSR calculated by multivariate Cox regression analysis showed the most discriminative effect of the TSR in grade III tumors compared to grade I and grade II tumors, and in triple‐negative tumors compared to nontriple‐negative tumors, for RFS and BCSS (Table 4). Kaplan–Meier analysis and log‐rank test for RFS of the TSR stratified by grade and triple‐negative status showed a statistically significant difference between subgroups (Figs. 1 and 2).

**Table 4:** Results of the tumor–stroma ratio stratified by clinically important prognostic parameters in the Nottingham Breast Cancer series and the multivariate Cox regression analysis per clinically relevant subgroups with a statistically significant difference

TSR stratified by group	Subgroups	Recurrence‐free survival	Breast cancer‐specific survival
Age		*p =* 0.881	*p =* 0.874
Tumor size		*p =* 0.422	*p =* 0.209
Grade		*p* < 0.001	*p =* 0.002
	Grade I	HR 1.16, 95% CI 0.58–2.29, *p* = 0.670	HR 6.34, 95% CI 0.81–49.95, *p* = 0.079
	Grade II	HR 0.78, 95% CI 0.55–1.10, *p* = 0.152	HR 0.83, 95% CI 0.54–1.30, *p* = 0.422
	Grade III	HR 1.89, 95% CI 1.43–2.51, *p* < 0.001	HR 1.86, 95% CI 1.35–2.57, *p* < 0.001
Histological type		*p =* 0.684	*p =* 0.951
ER status		*p =* 0.088	*p =* 0.101
PR status		*p =* 0.861	*p =* 0.532
HER2 status		*p =* 0.205	*p =* 0.851
Triple‐negative status		*p =* 0.040	*p =* 0.026
	Nontriple‐negative status	HR 1.21, 95% CI 0.97–1.51, *p* = 0.095	HR 1.27, 95% CI 0.96–1.67, *p* = 0.092
	Triple‐negative status	HR 1.86, 95% CI 1.10–3.14, *p* = 0.020	HR 2.24, 95% CI 1.24–4.07, *p* = 0.008
Lymph node status		*p =* 0.995	*p =* 0.432

Abbreviations: ER, estrogen receptor; HER2, human epidermal growth factor receptor 2; PR, progesterone receptor.

The ONCOPOOL study was used to validate the survival effects in grade III tumors and triple‐negative tumors. The interaction term for grade (*p* = 0.122) and triple‐negative status (*p* = 0.343) was not significant for RFS. The hazard ratio (HR) of the prognostic effect of the TSR for RFS were most discriminative in grade III tumors compared to grade I and grade II. If stratified by triple‐negative status, the HR of the TSR in nontriple‐negative tumors was lower compared to triple‐negative tumors, but this was not statistically significant. The interaction term was not statistically significant if stratified by age, tumor size, histology, ER status, PR status, HER2 status and lymph node status (Supporting Information Table S4).

## Discussion

In our study, we evaluated the prognostic value of TSR in, to the best of our knowledge, the largest cohort published on the prognostic value of TSR in breast cancer. The number of patients offered the opportunity to perform analyses of clinically relevant subgroups for breast cancer prognostication and treatment.

First, patients with a stroma‐high tumor had a worse prognosis compared to patients with a stroma‐low tumor. Second, the results of the Nottingham Breast Cancer series showed that the prognostic effect of the TSR was most discriminative in grade III tumors, compared to grade I and grade II tumors, and in triple‐negative tumors, compared to nontriple‐negative tumors. In the ONCOPOOL study, the HRs and confidence intervals of the TSR stratified by grade and triple‐negative status were comparable with the Nottingham Breast Cancer series. The interaction term showed no statistically significant effect for RFS if stratified by grade or triple‐negative status. A possible explanation of the lack of statistical significance is the moderate number of events.

Third, the prognostic effect of the TSR was not modified by age, tumor size, histology, ER status, PR status, HER2 status and lymph node status. This means that the prognostic value of the TSR in these clinically relevant subgroups does not differ from the prognostic value of the total cohort.

No former published literature has evaluated the prognostic value of the TSR by introducing the interaction term. Therefore, the results are not completely comparable. However, previous research showed higher HRs for the TSR in patients with triple‐negative tumors as overviewed by Kramer *et al*.^33^ The effect of the TSR stratified by grade has not been previously described.

The next step toward the clinical implementation of the TSR is to investigate the discriminating prognostic value of the TSR additional to the commonly used online PREDICT tool, which helps oncologists and patients in a shared decision toward personalized therapy.^37^, ^38^ Therefore, retrospective data will be analyzed and a prospective study such as the UNITED (Uniform Noting for International application of the TSRs as Easy Diagnostic Tool) study needs to be performed.^39^ The UNITED study is a prospective international multicenter study initiated by our research group. The study aim is to validate and prepare the TSR for implementation in standard clinical care in patients with colon cancer. Implementation of TSR assessment in standard clinical care has advantages compared to other potential biomarkers as this method is easy to perform, takes less than 2 min and requires no additional costs. Therefore, a comparable study for breast cancer would be desirable in the next step toward clinical implementation. Inter‐observer and intra‐observer reliability of the TSR assessment on digital slides in colon cancer is also evaluated in the UNITED study. The TSR assessment is explained to pathologists and residents *via* e‐learning and test sets.

The Nottingham Breast Cancer series is the first study in which the TSR is digitally assessed on breast cancer tissue by the method of Mesker *et al*.^7^ For the digital assessment, a field of 3.1 mm^2^ was used for final TSR scoring, which corresponds with conventional light microscopy used in our previous research. Van Pelt *et al*. described that the diameters of the different conventional light microscopes are between 2.54 and 3.80 mm^2^. However, this has not led to any major differences in the final score.^36^ One hundred percent of the slides were double scored in a blinded fashion by two observers (KV,WM), instead of the customary 30% double scoring, because of the possible learning curve of scoring digitally for the first time. The Cohen's kappa coefficient increased from 0.61 at first assessment to 0.87 in the second assessment of the slides with an incongruent value for the first time. In our opinion, observers who perform digital TSR assessment for the first time need to be aware of a learning curve. If this stage is passed, the TSR scoring on digital slides seems to be reliable and gives, therefore, a good perspective for further digital assessment.

Furthermore, the intratumoral stroma contains valuable prognostic information and may, therefore, be an important source for the development of new stroma based therapeutic agents. A major component of the tumor‐associated stroma and therefore a promising therapeutic target are cancer‐associated fibroblasts (CAFs). At the moment, CAFs are still difficult to target due to the lack of specific cell surface targets, as they are heterogeneous in phenotype and function. An important recent finding is the identification of CD10 and GPR77 as surface markers on CAFs in breast cancer. CD10^+^GPR77^+^ CAFs are predictive for response to chemotherapy and patient survival, particularly in breast tumors with a high grade.^40^ The authors showed that the disease‐free survival of breast cancer patients with a high CD10^+^GPR77^+^ CAF infiltration was significantly shorter. The disease‐free survival of patients with grade I and Grade II tumors was independent of CD10^+^GPR77^+^ CAF infiltration.^6^ These results are interesting as we found that the prognostic value of TSR is most discriminative in grade III tumors compared to grade I and grade II tumors. Whether CAF subtypes differ between stroma‐low and stroma‐high tumors is not known at this moment and requires further research.

Moreover, Ahn et al.^41^ concluded that, especially in patients with grade III tumors, the dominant stroma type was an independent risk factor for disease‐free survival in favor of patients with lymphocyte dominant stroma. Therefore, evaluation of the stromal composition would be interesting, for instance by dividing the stromal compartment in dominant stroma type; collagen, fibroblast or lymphocyte.

Advantages of our study are the large cohort size and long follow‐up period. A limitation of our study is the time period in which patients are included and as a consequence the changes in treatment modalities. In the studied patient groups, proportionally less patients received hormonal therapy than in current treatments. However, previously published research, including the ONCOPOOL study, showed that the TSR was of prognostic value in patients with hormone receptor‐positive tumors who received hormonal therapy.^9^, ^14^ This may suggest that the prognostic value of the TSR can be translated into current hormonal treatment strategies. Also, the introduction of Trastuzumab has positively influenced the clinical outcome. Therefore, a large, more recent retrospective study, in which the change in treatment modalities and a decent follow‐up period are considered, and/or a prospective cohort study should be performed to validate the TSR in the next step toward clinical implementation.

## Conclusions

The results showed that the prognostic effect of the TSR is most discriminative in grade III tumors, compared to grade I and grade II tumors, and in triple‐negative tumors, compared to nontriple‐negative tumors. Furthermore, the prognostic value of the TSR was not modified by age, tumor size, histology, ER status, PR status, HER2 status and lymph node status. This makes TSR a potential factor for inclusion to improve risk stratification. Validating the TSR in a prospective study could further improve clinical decision‐making using the PREDICT tool.

## Conflict of interest

The authors declare that there is no conflict of interest.

## Supporting information


**Figure S1** Representative tissue selection for tumor–stroma ratio assessment. (*a*) Stroma‐low, (*b*) Stroma‐high
**Figure S2** Kaplan–Meier analysis for recurrence‐free survival of patients included in the Nottingham Breast Cancer Series stratified by tumor–stroma ratio
**Table S1**. Overview of the stratification of age, tumor characteristics and treatment options of patients included in the ONCOPOOL study
**Table S2**. Univariate and multivariate Cox regression analysis of the ONCOPOOL study. The tumor–stroma ratio is adjusted for confounders
**Table S3**. Results of the independent prognostic value of the tumor–stroma ratio adjusted for confounders, triple‐negative status and lymph node status calculated with multivariate Cox regression analysis in the ONCOPOOL study
**Table S4**. Results of tumor–stroma ratio stratified by clinically important prognostic parameters in the ONCOPOOL study and the multivariate Cox regression analysis per clinically relevant subgroup with a statistically significant difference in the Nottingham Breast Cancer series.

## Data Availability

Data are available on reasonable request.
