# Supplementary material for: The prognostic value of the tumor–stroma ratio is most discriminative in patients with grade III or triple‐negative breast cancer
Source: Int J Cancer. 2020 Jan 22;146(8):2296–304. doi: 10.1002/ijc.32857 (PMC7065011; doi:10.1002/ijc.32857)
Supplement: Supplementary file 1 — Figure S1 Representative tissue selection for tumor–stroma ratio assessment. (a) Stroma‐low, (b) Stroma‐high Figure S2 Kaplan–Meier analysis for recurrence‐free survival of patients included in the Nottingham Breast Cancer Series stratified by tumor–stroma ratio Table S1. Overview of the stratification of age, tumor characteristics and treatment options of patients included in the ONCOPOOL study Table S2. Univariate and multivariate Cox regression analysis of the ONCOPOOL study. The tumor–stroma ratio is adjusted for confounders Table S3. Results of the independent prognostic value of the tumor–stroma ratio adjusted for confounders, triple‐negative status and lymph node status calculated with multivariate Cox regression analysis in the ONCOPOOL study Table S4. Results of tumor–stroma ratio stratified by clinically important prognostic parameters in the ONCOPOOL study and the multivariate Cox regression analysis per clinically relevant subgroup with a statistically significant difference in the Nottingham Breast Cancer series. [file IJC-146-2296-s001.pdf]

Supplementary figures and tables of the manuscript titled:

The prognostic value of the tumor-stroma ratio is most discriminative in patients with grade III or triple negative breast cancer.

Kiki M.H. Vangangelt, Andrew R. Green, Isabelle (M) F. Heemskerk, Danielle Cohen, Gabi W. van Pelt, Marcelo Sobral-Leite, Marjanka K. Schmidt, Hein Putter, Emad A. Rakha, Rob A.E.M. Tollenaar, Wilma E. Mesker

Table of Contents of supplementary figures and tables

- Supplementary figure 1 p.3
- Supplementary figure 2 p.4
- Supplementary table 1 p.5
- Supplementary table 2 p.7
- Supplementary table 3 p.9
- Supplementary table 4 p.10

Supplementary figure 1. Representative tissue selection for tumor-stroma ratio assessment. **a.** Stroma-low, **b.** Stroma-high.

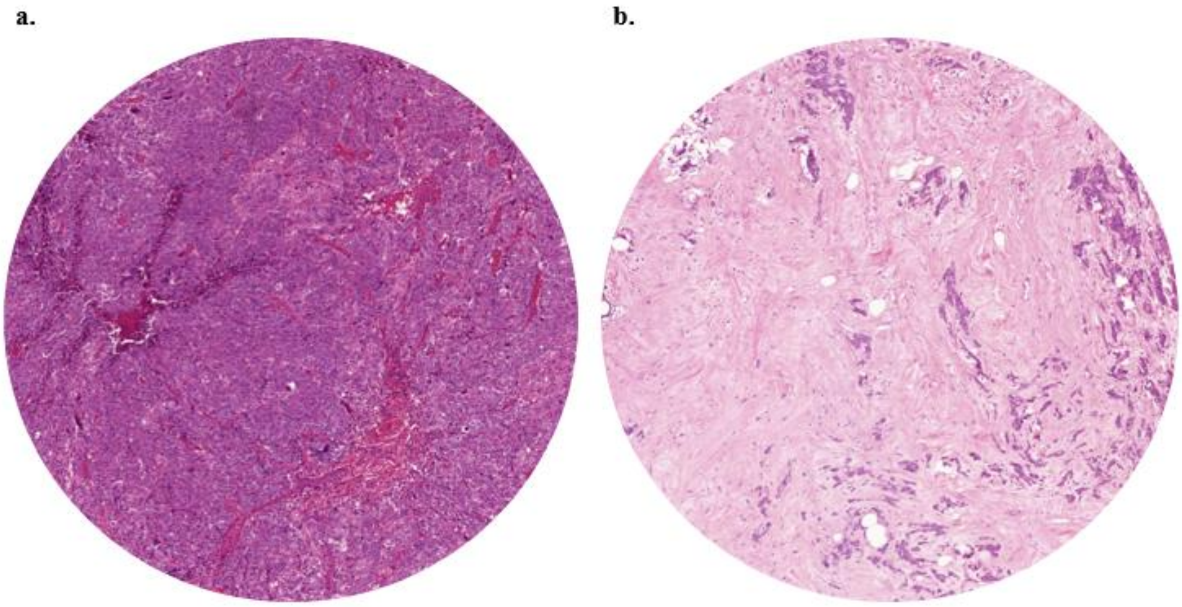

Supplementary figure 2. Kaplan-Meier analysis for recurrence-free survival of patients included in the Nottingham Breast Cancer Series stratified by tumor-stroma ratio.

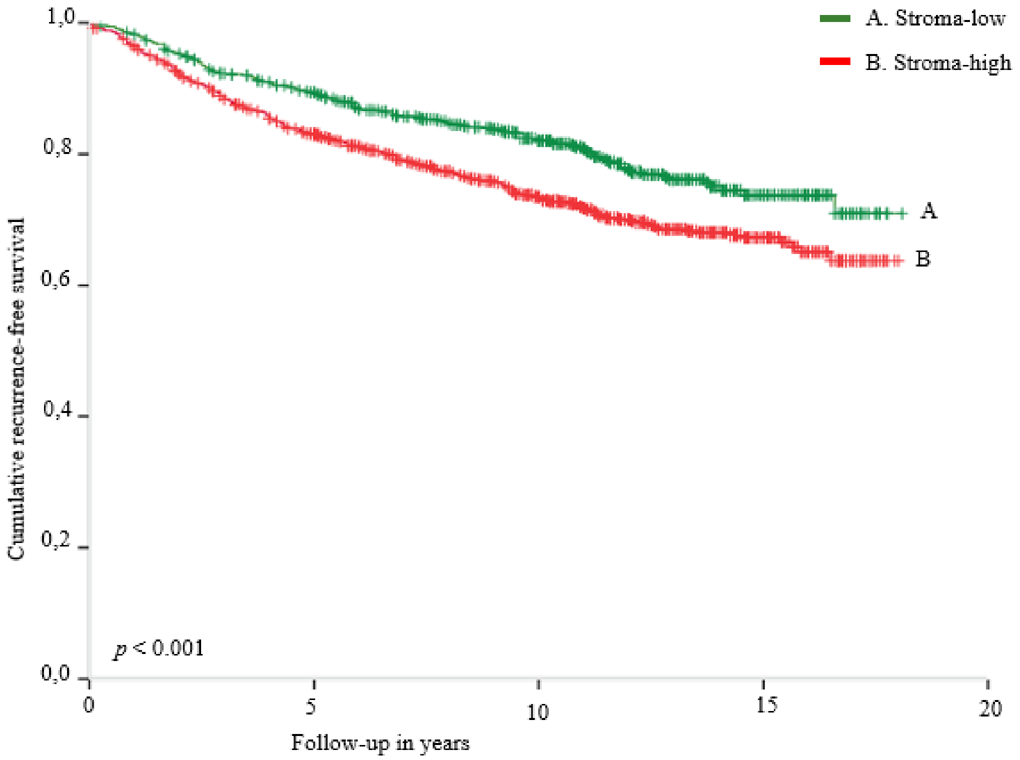

Numbers at risk

Stroma-low	681	565	372	70	9
Stroma-high	1113	858	564	105	15

Supplementary table 1. Overview of the stratification of age, tumor characteristics and treatment options of patients included in the ONCOPOOL study.

		**Stroma-low**		**Stroma-high**		
	n	n = 454	%	n = 283	%	*p*-value
Age (in years)						
<40	63	49	10.8	14	4.9	0.012
40-49	206	134	29.5	72	25.4	
50-59	259	147	32.4	112	39.6	
≥60	209	124	27.3	85	30.0	
Histological type						
Invasive carcinoma of NST	621	386	85.0	235	83.0	0.484
Lobular carcinoma	69	38	8.4	31	11.0	
Tubular carcinoma	32	22	4.8	10	3.5	
Others	15	8	1.8	7	2.5	
Grade						
I	159	101	22.2	58	20.5	0.274
II	255	146	32.2	109	38.5	
III	216	142	31.3	74	26.1	
Missing	107	65	14.3	42	14.8	
Tumor size (in cm's)						
≤2	479	314	69.2	165	58.3	0.005
>2-≤5	252	135	29.7	117	41.3	
>5	6	5	1.1	1	0.4	
Nodal status						
Negative	416	257	56.6	159	56.2	0.145
Positive	315	191	42.1	124	43.8	
Missing	6	6	1.3	0	0	
ER status						
Negative	127	87	19.2	40	14.1	0.142
Positive	606	365	80.4	241	85.2	
Missing	4	2	0.4	2	0.7	
PR status						
Negative	234	149	32.8	85	30.0	0.442
Positive	496	302	66.5	194	68.6	
Missing	7	3	0.7	4	1.4	
HER2 status						
Negative	573	357	78.6	216	76.3	0.462
Positive	151	91	20.0	60	21.2	
Missing	13	6	1.3	7	2.5	
Chemotherapy						
No	573	346	76.2	227	80.2	0.204
Yes	164	108	23.8	56	19.8	
Hormonal therapy						
No	369	229	50.4	140	49.5	0.798

Yes	368	225	49.6	143	50.5	

Abbreviations: ER = estrogen receptor, HER2 = human epidermal growth factor receptor 2, NST = no special type, PR = progesterone receptor

Supplementary table 2. Univariate and multivariate Cox regression analysis of the ONCOPOOL study. The tumor-stroma ratio is adjusted for confounders.

		**Recurrence-free survival**						**Overall survival**					
		Univariate analysis			*Multivariate analysis*			*Univariate analysis*			*Multivariate analysis*		
	n	HR	95% CI	*p*-value	HR	95% CI	*p*-value	HR	95% CI	*p*-value	HR	95% CI	*p*-value
Age													
	733	1.00	0.98 to 1.01	0.482	1.00	0.98 to 1.02	0.978	1.04	1.03 to 1.05	<0.001	1.04	1.03 to 1.06	<0.001
Tumor size (in cm's)													
≤2	479			<0.001			0.098			<0.001			0.028
>2	258	1.77	1.35 to 2.32		1.31	0.95 to 1.79		1.65	1.30 to 2.09		1.37	1.04 to 1.82	
Grade													
I	159			<0.001			0.022			<0.001			0.001
II	255	1.86	1.22 to 2.86		1.63	1.04 to 2.56		1.64	1.13 to 2.38		1.65	1.10 to 2.46	
III	216	2.51	1.64 to 3.85		2.00	1.22 to 3.28		2.47	1.71 to 3.56		2.24	1.46 to 3.43	
Histological type													
Invasive carcinoma of NST	621			0.657			0.464			0.461			0.326
Lobular carcinoma	69	0.99	0.63 to 1.56		1.32	0.74 to 2.35		0.93	0.62 to 1.40		1.11	0.63 to 1.97	
Tubular carcinoma	32	0.69	0.32 to 1.47		0.92	0.39 to 2.15		0.93	0.53 to 1.63		1.02	0.52 to 2.00	
Others	14	0.61	0.19 to 1.91		0.41	0.10 to 1.67		0.40	0.13 to 1.26		0.16	0.02 to 1.15	
ER status													
Negative	127			<0.001			0.542			<0.001			0.470
Positive	606	0.55	0.40 to 0.76		0.87	0.55 to 1.37		0.54	0.41 to 1.71		0.87	0.59 to 1.27	
PR status													
Negative	234			<0.001			0.271			<0.001			0.016
Positive	496	0.60	0.45 to 0.79		0.81	0.55 to 1.18		0.51	0.40 to 0.64		0.67	0.49 to 0.93	
HER2 status													
Negative	573			0.572			0.896			0.017			0.033
Positive	151	1.10	0.79 to 1.53		1.02	0.72 to 1.47		1.40	1.06 to 1.84		1.39	1.03 to 1.88	

TSR													
Stroma-low	454			0.093			0.085			0.016			0.029
Stroma-high	283	1.26	0.96 to 1.66		1.30	0.96 to 1.76		1.34	1.06 to 1.69		1.35	1.03 to 1.77	

Abbreviations: ER = estrogen receptor, HER2= human epidermal growth factor receptor 2, NST = no special type, PR = progesterone receptor, TSR= tumor-stroma ratio

Supplementary table 3. Results of the independent prognostic value of the tumor-stroma ratio adjusted for confounders, triple-negative status and lymph node status calculated with multivariate Cox regression analysis in the ONCOPOOL study.

	**Recurrence-free survival**	**Overall survival**
Confounders	HR 1.30, 95% CI 0.96 to 1.76, *p* = 0.085	HR 1.35, 95% CI 1.03 to 1.77, *p* = 0.029
Confounders including triple-negative status	HR 1.30, 95% CI 0.96 to 1.75, *p* = 0.091	HR 1.34, 95% CI 1.02 to 1.74, *p* = 0.033
Confounders and lymph node status	HR 1.28, 95% CI 0.95 to 1.73, *p* = 0.112	HR 1.33, 95% CI 1.02 to 1.74, *p* = 0.037

Supplementary table 4. Results of tumor-stroma ratio stratified by clinically important prognostic parameters in the ONCOPOOL study and the multivariate Cox regression analysis per clinically relevant subgroup with a statistically significant difference in the Nottingham Breast Cancer series.

**TSR stratified by group**	**Subgroups**	**Recurrence-free survival**	**Overall survival**
Age		p = 0.496	p = 0.840
Tumor size		p = 0.816	p = 0.823
Grade		p = 0.122	p = 0.414
	Grade I	HR 0.92, 95% CI 0.39 to 2.35, p = 0.992	HR 0.83, 95% CI 0.38 to 1.81, p = 0.631
	Grade II	HR 1.06, 95% CI 0.66 to 1.70, p = 0.806	HR 1.30, 95% CI 0.83 to 2.02, p = 0.257
	Grade III	HR 1.86, 95% CI 1.18 to 2.93, p = 0.008	HR 1.61, 95% CI 1.08 to 2.41, p = 0.020
Histological type		p = 0.838	p = 0.620
ER status		p = 0.445	p = 0.222
PR status		p = 0.982	p = 0.387
HER2 status		p = 0.646	p = 0.910
Triple-negative status		p = 0.343	p = 0.255
	Non-triple-negative status	HR 1.25, 95% CI 0.91 to 1.73, p = 0.176	HR 1.28, 95% CI 0.96 to 1.72, p = 0.093
	Triple-negative status	HR 1.54, 95% CI 0.64 to 3.66, p = 0.333	HR 1.75, 95% CI 0.83 to 3.66, p = 0.140
Lymph node status		p = 0.423	p = 0.097

Abbreviations: ER = estrogen receptor, HER2 = human epidermal growth factor receptor, PR = progesterone receptor
